# In vitro fertilization outcome based on the detailed early luteal phase trajectory of hormones: a prospective cohort study

**DOI:** 10.1186/s12958-024-01229-3

**Published:** 2024-05-20

**Authors:** Lan N Vuong, Toan D Pham, Vu N A Ho, Anh T L Vu, Tuong M Ho, Claus Yding Andersen

**Affiliations:** 1 Department of Obstetrics and Gynaecology, University of Medicine and Pharmacy at Ho Chi Minh City, 217 Hong Bang Street, District 5, Ho Chi Minh City, Vietnam; 2 IVFMD, My Duc Hospital, Ho Chi Minh City, Vietnam; 3 HOPE Research Center, My Duc Hospital, Ho Chi Minh City, Vietnam; 4 Institute of Clinical Medicine, The Faculty of Health Science, Copenhagen University, Copenhagen, Denmark

**Keywords:** In vitro fertilization, Infertility, Luteal phase support, Live birth, Progesterone, Hormone levels

## Abstract

**Background:**

Ovarian stimulation and the use of human chorionic gonadotropin (hCG) for triggering oocyte maturation in women undergoing in vitro fertilisation (IVF) introduces several differences in luteal phase hormone levels compared with natural cycles that may negatively impact on endometrial receptivity and pregnancy rates after fresh embryo transfer. Exogenous luteal phase support is given to overcome these issues. The suitability of a pragmatic approach to luteal phase support is not known due to a lack of data on early phase luteal hormone levels and their association with fertility outcomes during IVF with fresh embryo transfer. This study determined early luteal phase profiles of serum progesterone, 17-hydroxyprogesterone and hCG, and associations between hormone levels/hormone level profile after hCG trigger and the live birth rate in women undergoing IVF with fresh embryo transfer.

**Methods:**

This prospective single center, cohort study was conducted in Vietnam from January 2021 to December 2022. Women aged 18–38 years with normal ovarian reserve and undergoing controlled ovarian stimulation using a gonadotropin-releasing hormone antagonist protocol were included. Serum hormone levels were determined before trigger, at 12, 24 and 36 h after hCG, and daily from 1 to 6 days after oocyte pick-up. Serum hormone level profiles were classified as lower or upper. The primary outcome was live birth rate based on early luteal phase hormone level profile.

**Results:**

Ninety-five women were enrolled. Live birth occurred in 19/69 women (27.5%) with a lower progesterone profile and 13/22 (59.1%) with an upper progesterone profile (risk ratio [RR] 2.15; 95% confidence interval [CI] 1.28–3.60), and in 6/31 (19.4%) versus 26/60 (43.3%) with a lower versus upper serum 17-hydroxyprogesterone profile (RR 2.24; 95% CI 1.03–4.86). Nearly 20% of women had peak progesterone concentration on or before day 3 after oocyte pick-up, and this was associated with significantly lower chances of having a life birth.

**Conclusions:**

These data show the importance of proper corpus luteum function with sufficient progesterone/17-hydroxyprogesterone production for achievement of pregnancy and to maximize the chance of live birth during IVF.

**Trial Registration:**

NCT04693624 (www.clinicaltrials.gov).

**Supplementary Information:**

The online version contains supplementary material available at 10.1186/s12958-024-01229-3.

## Introduction

The use of human chorionic gonadotropin (hCG) for triggering of oocyte maturation in women undergoing in vitro fertilization (IVF) treatment has been considered the gold standard approach since IVF was introduced four decades ago. The hCG trigger serves two main functions: advancement of oocyte meiosis to the metaphase of the second division ready for fertilization and further development, and stimulating the corpora lutea to secrete progesterone during the early luteal phase due to its relatively long half-life [1–3].

There are, however, several differences in luteal phase hormone levels compared with natural cycles when using hCG for ovulation induction, which may negatively impact on endometrial receptivity and chances of pregnancy. These differences include an immediate rise in serum progesterone concentration, substantially higher maximal luteinizing hormone-like activity (there is no hCG in a natural cycle), higher progesterone concentrations, and earlier timing of peak serum progesterone level [4–6]. Furthermore, at the time of implantation (6 to 7 days after oocyte pick-up), hCG has almost been eliminated and the drive for progesterone synthesis reduced. The current approach to improve luteal phase functioning is to administer exogenous hormones for luteal phase support but this only increases serum progesterone concentrations to a limited extent. Furthermore, luteal phase support is currently given using a “one size fits all” approach.

The absence of evidence-based individualized luteal phase support is partly due to the lack of detailed information on how the pattern of corpora lutea-secreted hormones affects pregnancy and live birth rates. Furthermore, it is not yet known whether information on corpora lutea hormone profiles shortly after administration of hCG trigger for final oocyte maturation can provide predictive information on the hormone level profiles during the luteal phase and be used to adjust and improve the luteal phase support given, with a potential increase in the clinical pregnancy rate.

This study determined detailed profiles of serum progesterone, 17-hydroxyprogesterone and hCG levels in the early luteal phase until the time of implantation and related this to live birth rates in women undergoing IVF with hCG trigger followed by fresh embryo transfer.

## Method

### Study design

This prospective, single-center, cohort study (NCT04693624) was conducted at IVFMD, My Duc Hospital, Vietnam from January 2021 to December 2022. The study protocol was approved by the hospital’s Ethic Review Board (approval number: 18/20/DD-BVMD). Study procedures were conducted in accordance with Good Clinical Practice and Declaration of Helsinki principles. All participants provided written informed consent.

### Study population

Eligible women were aged 18–38 years with an indication for IVF with hCG trigger for final oocyte maturation and fresh embryo transfer, and had the following: body mass index < 28 kg/m^2^; normal ovarian reserve (anti-Müllerian hormone level > 8.93 pmol/L or antral follicle count ≥ 6 within two months before stimulation); 4–19 follicles with a diameter of ≥ 14 mm on the day of hCG triggering; and standard gonadotropin-releasing hormone antagonist protocol for ovarian stimulation. Individuals with a previous poor ovarian response (≤ 3 follicles after appropriate follicle-stimulating hormone stimulation), hyper-response (20 follicles of ≥ 14 mm in diameter) on the day of trigger, polycystic ovary syndrome, chronic medical conditions, and/or participating in other clinical trials were excluded.

### IVF protocol

Standard clinic procedures were used for stimulation, monitoring and oocyte pick-up. In brief, subcutaneous administration of recombinant follicle-stimulating hormone was started on cycle day 2 at 150–300 IU/day based on the serum anti-Müllerian hormone level. Subcutaneous gonadotropin-releasing hormone antagonist 0.25 mg/day was administered from day 5 of stimulation. When ≥ 2 follicles reached ≥ 17 mm in diameter, subcutaneous hCG 6,500 IU (Ovitrelle, Merck Serono, Italy) was injected. All individuals had transfer of a maximum of two fresh day-3 embryos; any remaining embryos were frozen for later use.

Luteal phase support consisted of vaginal progesterone (Cyclogest, Actavis) 400 mg twice daily starting on the day of oocyte pick-up and continued until pregnancy testing at 2 weeks after embryo transfer (Cobas, Roche, Diagnostics), then continued until 7 weeks’ gestation if the pregnancy test was positive (beta hCG > 5 mIU/mL).

### Assessments

Blood samples (2 mL each) for analysis of serum concentrations of progesterone, 17-hydroxyprogesterone and hCG were collected on the day of triggering (before hCG), at 12, 24 and 36 h after hCG administration, and at 1, 2, 3, 4, 5 and 6 days after oocyte pick-up. Serum progesterone concentration reflects hormone secreted by the corpus luteum and exogenous vaginal progesterone used for luteal phase support, while serum 17-hydroxyprogesterone reflects only corpus luteum production.

Concentrations of progesterone, 17-hydroxyprogesterone and hCG were determined at the Clinical Biochemistry department at My Duc Hospital using Elecsys progesterone III (Germany) for progesterone measurement, 17-hydroxyprogesterone ELISA (Germany) for 17-hydroxyprogesterone measurement and ARCHITECT Total β-hCG Reagent Kit (Ireland) for β-hCG measurement. The standard and validated assays used had negligible cross-reactivity between progesterone and 17-hydroxyprogesterone determinations.

### Outcomes

The primary outcome was live birth rate in relation to the early luteal phase serum hormone level profile. Live birth was defined as the birth of at least one newborn after 24 weeks’ gestation that exhibited any sign of life (twins were a single count).

### Statistical analysis

Shapiro-Wilk test was used to check for normal distribution of quantitative variables. Data are presented as mean ± standard deviation (normal distribution) or median [interquartile range] (skewed distribution).

Continuous and categorical variables were analyzed using descriptive analysis (mean ± standard deviation, median [interquartile range], or number and percentage). Baseline characteristics associated with live birth outcomes were first examined using univariate and multivariate regression models. Following this, the K-means clustering method, with a specified cluster count of 2, was applied for the categorization of test points into distinct profiles. This method, an unsupervised algorithm, facilitates the organization of unlabeled data into clusters through the initial selection of centroids, the assignment of data points to the nearest cluster, and the iterative updating of centroids until the cluster assignments remain stable, effectively forming two distinct clusters. The influence of individual hormone levels and combined hormone profiles on the likelihood of live birth was then investigated, with the adjusted odds ratio and 95% confidence interval (CI) values for anti-Müllerian hormone levels being assessed. Finally, a post-hoc analysis was conducted to compare individual and cycle characteristics between participants with lower versus upper profiles of serum progesterone and 17-hydroxyprogesterone, aiming to identify associated factors of distinct hormone profiles.

Statistical analyses were performed using the R statistical program, version 4.3.0. A p-value of < 0.05 was defined as statistically significant.

## Results

### Study population

Ninety-five women were enrolled (Table 1). Over 82% were undergoing their first IVF cycle. The most common indications for IVF were male factors and unexplained infertility. Mean duration of ovarian stimulation was 9 days (range 6–12 days). The results of ovarian stimulation (Table 1) were consistent with clinical practice at our center. There were no instances of ovarian hyperstimulation syndrome.

**Table 1:** Patient and cycle characteristics at baseline

Characteristic	Participants (*n* = 95)*
Age, years	32.8 ± 3.9
Body mass index, kg/m^2^	21.4 ± 2.8
Anti-Müllerian hormone, pmol/L	14.43 [9.21; 23.25]
Antral follicle count, n	13.0 [8.0; 17.0]
Duration of infertility, years	3.0 [1.8; 4.3]
Type of infertility, n (%)	
Primary	60 (63.2)
Secondary	35 (36.8)
Number of previous IVF/ICSI cycles, n (%)	
1	78 (82.1)
2	15 (15.8)
3	2 (2.1)
Indication for IVF, n (%)	
Unexplained infertility	39 (41.1)
Male factor infertility	29 (30.5)
Tubal factor infertility	10 (10.5)
Advance female age	6 (6.3)
Other	11 (11.6)
Total dose of follicle-stimulating hormone, IU	2400.0 [2025.0; 2700.0]
Number of follicles ≥ 14 mm	7.0 [4.0; 9.0]
Estradiol on day of trigger, pg/mL	2443.0 [1433.5; 3700.8]
Number of oocytes	10.0 [6.0; 13.0]
Number of metaphase II oocytes	8.0 [4.5; 10.0]
Number of fertilized oocytes	5.0 [3.0; 7.5]
Number of day 3 embryos	5.0 [3.0; 7.0]
Number of good^#^ day 3 embryos	3.0 [2.0; 6.0]
Number of frozen embryos	2.0 [0.0; 4.0]
Number of embryos transferred	
1	9 (9.5)
2	86 (90.5)
Number of good^#^ embryos transferred	
0	5 (5.5)
1	16 (17.6)
2	70 (76.9)
Positive pregnancy test, n (%)	47 (49.5)
Clinical pregnancy, n (%)	41 (43.2)
Ongoing pregnancy, n (%)	33 (34.7)
Live birth, n (%)	33 (34.7)
Miscarriage, n (%)	7 (7.4)
Ectopic pregnancy, n (%)	1 (1.1)

Values are mean ± standard deviation, median [interquartile range], or number of participants (%)

*Data for anti-Müllerian hormone were available in 83 participants, antral follicle count in 88 participants, and duration of infertility in 79 participants; all other parameters had data available for all 95 participants

^#^According to the Istanbul consensus

### Early luteal phase hormone profiles

**Fig. 1:**
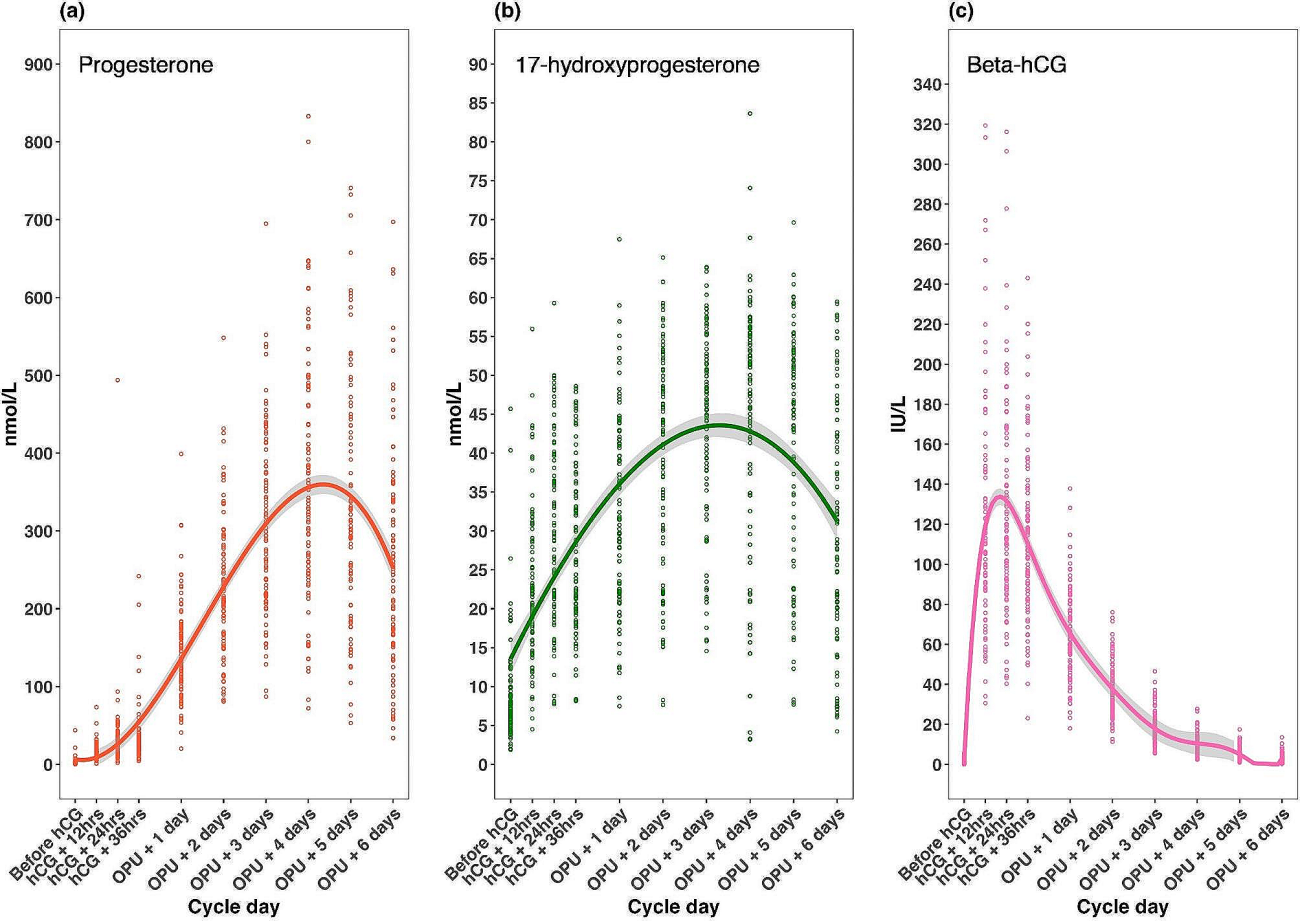
Hormone level profiles for progesterone **(a)**, 17-hydroxyprogesterone **(b)** and beta-human chorionic gonadotropin (beta-hCG) **(c)** in the early luteal phase after human chorionic gonadotropin (hCG) trigger. *OPU* oocyte pick-up

Serum progesterone and 17-hydroxyprogesterone levels started to increase on the day after hCG administration, peaking four days after oocyte pick up (at 366 ± 16 nmol/L and 44 ± 1.6 nmol/L, respectively) then starting to decrease. The highest serum hCG level was 126 mIU/mL, observed 24 h after hCG trigger, followed by a gradual decline (Fig. 1, Table S1).

Out of 95 women, 91 had all ten blood samples collected, three missed the sample at OPU + 6 days and one missed the sample before hCG. Therefore, only 91 women were clustered into the lower and upper hormonal profiles when performing K-means clustering. Sixty-nine women had a lower serum progesterone profile, 22 had an upper serum progesterone profile, 31 had a lower 17-hydroxyprogesterone profile, 60 had an upper 17-hydroxyprogesterone profile, 47 had lower hCG profile, and 44 had an upper hCG profile. Serum hormone levels over time in these subgroups are shown in Table S2. Several clinical and cycle characteristics differed significantly between individuals with a lower versus upper serum profile of progesterone or 17-hydroxyprogesterone, including the anti-Müllerian hormone level and antral follicle count (Tables 2 and 3).

**Table 2:** Factors associated with a lower serum progesterone level profile

Characteristic	Lower profile group (*n* = 69)	Upper profile group (*n* = 22)	OR (95% CI), *p*-value	
			Univariate	Multivariate
Age, years	32·8 ± 3·9	33·0 ± 4·1	0.99 [0.88; 1.12], 0.892	-
Body mass index, kg/m^2^	21·4 ± 2·8	21·4 ± 2·3	1.01 [0.84; 1.21], 0.947	-
Anti-Müllerian hormone, pmol/L	15·34 ± 9·83	26·50 ± 11·89	0.93 [0.90; 0.97], 0.001	0.88 [0.79, 0.96], 0.009
Antral follicle count, n	10.8 ± 4.0	14.1 ± 3.8	0.81 [0.71; 0.93], 0.002	1.07 [0.84, 1.40], 0.592
Duration of infertility, years	4.0 ± 3.3	4.2 ± 4.2	0.98 [0.86; 1.13], 0.825	-
Type of infertility, n (%)				
Primary	44 (63·8)	13 (59·1)	Ref.	-
Secondary	25 (36·2)	9 (40·9)	0.82 [0.31; 2.27], 0.696	-
Number of previous IVF/ICSI cycles, n (%)				
1	53 (76·8)	21 (95·5)	Ref.	-
2	14 (20·3)	1 (4·6)	-	-
3	2 (2·9)	0 (0·0)	-	-
Total dose of follicle-stimulating hormone, IU	2459.8 ± 698.3	2181.8 ± 488.8	1.00 [1.00; 1.00], 0.089	-
Number of follicles ≥ 14 mm	6.2 ± 3.2	10.8 ± 5.2	0.69 [0.57; 0.84], < 0.001	1.00 [1.00, 1.00], 0.915
Number of oocytes	8.6 ± 4.4	14.2 ± 5.8	0.79 [0.70; 0.90], < 0.001	0.66 [0.46, 0.86], 0.013
Number of metaphase II oocytes	6.7 ± 3.6	11.1 ± 4.9	0.77 [0.67; 0.89], < 0.001	0.83 [0.53, 1.26], 0.379
Number of fertilized oocytes	4.9 ± 3.3	8.4 ± 3.9	0.77 [0.66; 0.89], < 0.001	0.78 [0.41, 1.41], 0.433
Number of day 3 embryos	4.6 ± 2.8	7.9 ± 3.8	0.74 [0.62; 0.87], < 0.001	2.18 [1.17, 4.55], 0.021
Number of good* day 3 embryos	3.7 ± 2.7	5.8 ± 3.5	0.80 [0.67; 0.94], 0.007	0.31 [0.11, 0.70], 0.01
Number of frozen embryos	1.9 ± 1.9	3.6 ± 1.9	0.65 [0.50; 0.85], 0.001	2.37 [1.10, 6.38], 0.046
Number of embryos transferred				
1	9 (13.0)	0 (0.0)	-	-
2	60 (87.0)	22 (100.0)	-	-
Number of good* embryos transferred				
≤ 1	20 (29.0)	1 (4.5)	Ref.	Ref.
2	49 (71.0)	21 (95.5)	0.13 [0.01; 0.71], 0.014	0.16 [0.00, 2.93], 0.281

Values are mean ± standard deviation, median [interquartile range] or number of participants (%)

*According to the Istanbul consensus

**Table 3:** Factors associated with a lower serum 17-hydroxyprogesterone level profile

Characteristic	Lower profile group (*n* = 31)	Upper profile group (*n* = 60)	OR (95% CI), *p*-value	
			Univariate	Multivariate
Age, years	33·5 ± 4·2	32·5 ± 3·7	1.07 [0.95; 1.19], 0.271	-
Body mass index, kg/m^2^	21·7 ± 2·4	21·3 ± 2·8	1.06 [0.90; 1.25], 0.469	-
Anti-Müllerian hormone, pmol/L	11·48 ± 8·57	21·24 ± 11·22	0.91 [0.86; 0.96], 0.001	0.97 [0.90, 1.03], 0.346
Antral follicle count, n	9.23 ± 3.73	12.78 ± 3.88	0.79 [0.69; 0.90], < 0.001	0.98 [0.81, 1.20], 0.841
Duration of infertility, years	2·0 [1·3; 4·5]	3·0 [2·0; 4·6]	1.03 [0.92; 1.17], 0.589	-
Type of infertility, n (%)				
Primary	20 (64·5)	37 (61·7)	Ref.	-
Secondary	11 (35·5)	23 (38·3)	0.89 [0.35; 2.19], 0.8	-
Number of previous IVF/ICSI cycles, n (%)				
1	23 (74·2)	51 (85·0)	Ref.	-
2	7 (22·6)	8 (13·3)	0.52 [0.16; 1.67], 0.266	-
3	1 (3·2)	1 (1·7)	0.46 [0.01; 18.3], 0.632	-
Total dose of follicle-stimulating hormone, IU	2637.1 ± 807.8	2266.2 ± 538.0	1.00 [1.00; 1.00], 0.016	1.00 [1.00, 1.00], 0.103
Number of follicles ≥ 14 mm	4.5 ± 2.9	8.8 ± 4.1	0.63 [0.52; 0.78], < 0.001	0.71 [0.51, 0.93], 0.026
Number of oocytes	6.1 ± 3.7	12.0 ± 4.9	0.70 [0.60; 0.82], < 0.001	0.67 [0.42, 1.01], 0.066
Number of metaphase II oocytes	4.7 ± 2.8	9.3 ± 4.3	0.66 [0.55; 0.80], < 0.001	0.80 [0.46, 1.34], 0.401
Number of fertilized oocytes	3.5 ± 2.3	6.9 ± 3.8	0.70 [0.58; 0.85], < 0.001	0.87 [0.42, 1.74], 0.686
Number of day 3 embryos	3.5 ± 2.3	6.3 ± 3.4	0.69 [0.56; 0.85], < 0.001	0.85 [0.25, 2.41], 0.772
Number of good* day 3 embryos	2.9 ± 2.6	4.8 ± 3.1	0.77 [0.63; 0.93], 0.006	3.53 [1.52, 10.22], 0.008
Number of frozen embryos	1.3 ± 1.5	2.8 ± 2.1	0.63 [0.48; 0.84], 0.002	0.60 [0.23, 1.53], 0.281
Number of embryos transferred				
1	6 (19.4)	3 (5.0)	Ref.	Ref.
2	25 (80.6)	57 (95.0)	0.23 [0.04; 0.97], 0.045	1.76 [0.08, 45.57], 0.717
Number of good* embryos transferred				
≤ 1	13 (41.9)	8 (13.3)	Ref.	Ref.
2	18 (58.1)	52 (86.7)	0.22 [0.07;0.61], 0.003	0.42 [0.02, 5.70], 0.53

Values are mean ± standard deviation, median [interquartile range] or number of participants (%)

*According to the Istanbul consensus

### Live birth rate

**Fig. 2:**
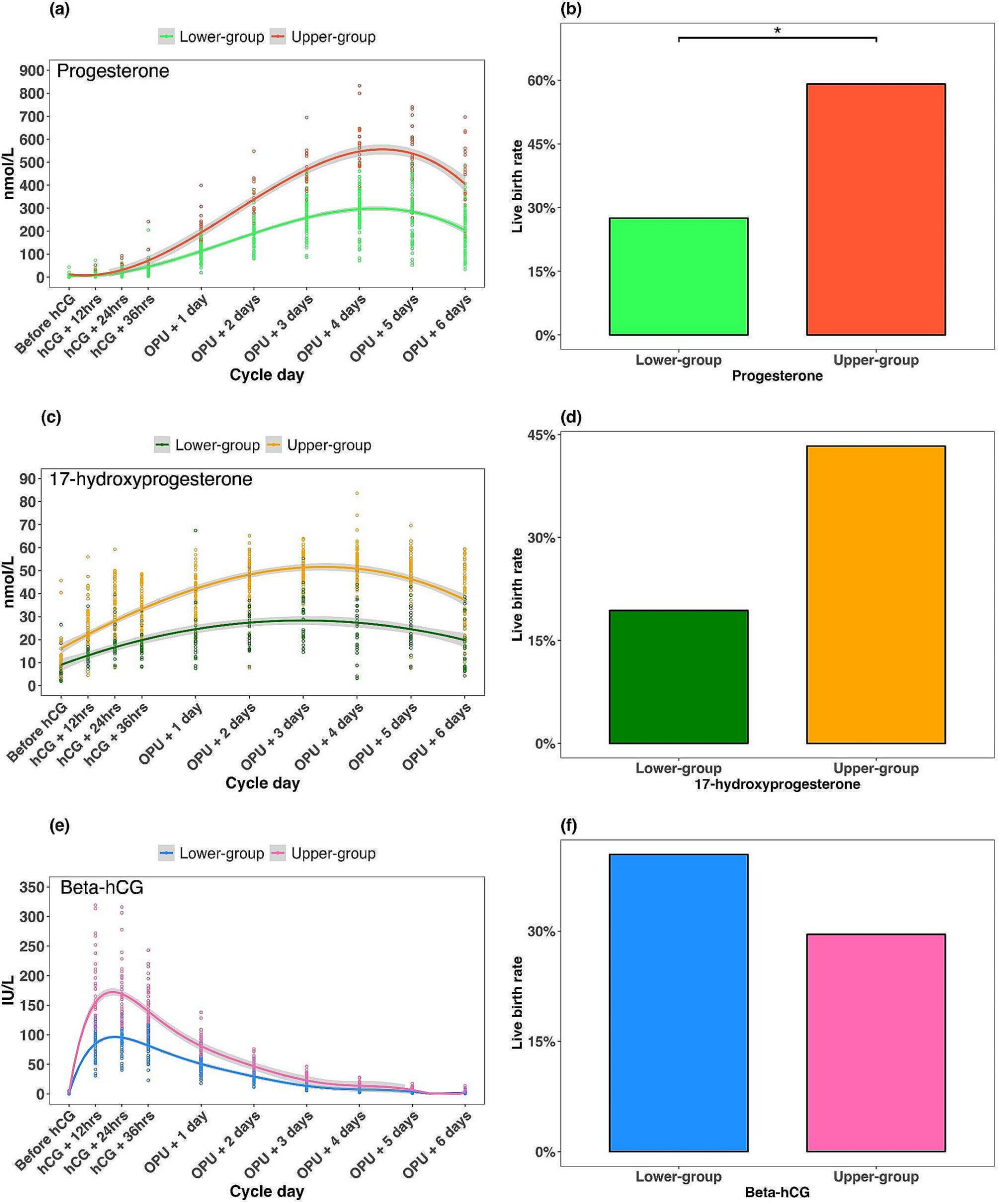
Lower and upper hormone profiles over time for serum progesterone **(a)**, serum 17-hydroxyprogesterone **(c)** and serum beta-human chorionic gonadotropin (hCG) **(e)**; and comparative rates of live birth in individuals with a lower versus upper profile of serum progesterone **(b)**, serum 17-hydroxyprogesterone **(d)**, or serum beta-hCG **(f)**. Shaded areas either side of the hormone profile lines indicate the upper and lower bounds of the 95% confidence interval. *adjusted p-value < 0.05

There was no correlation between serum levels of progesterone, 17-hydroxy progesterone or beta hCG and the live birth at each time point in analyses adjusted for the baseline anti-Müllerian hormone level (Table S3) and for the number of good embryos transferred (Table S4). However, using the K-mean method with number of clusters of two, we looked at data from the ten different time points comprehensively to create one profile of progesterone, 17-hydroxyprogesterone and beta hCG levels during early luteal phase. This analysis showed that there was a significantly higher live birth rate in the upper versus lower serum progesterone profile group, no significant difference in the live birth rate between the upper versus lower serum 17-hydroxyprogesterone group, and the upper and lower serum beta hCG groups (Fig. 2). There was a significant correlation between the hormonal profile for serum progesterone and the live birth rate (Table S3, Table S4).

**Fig. 3:**
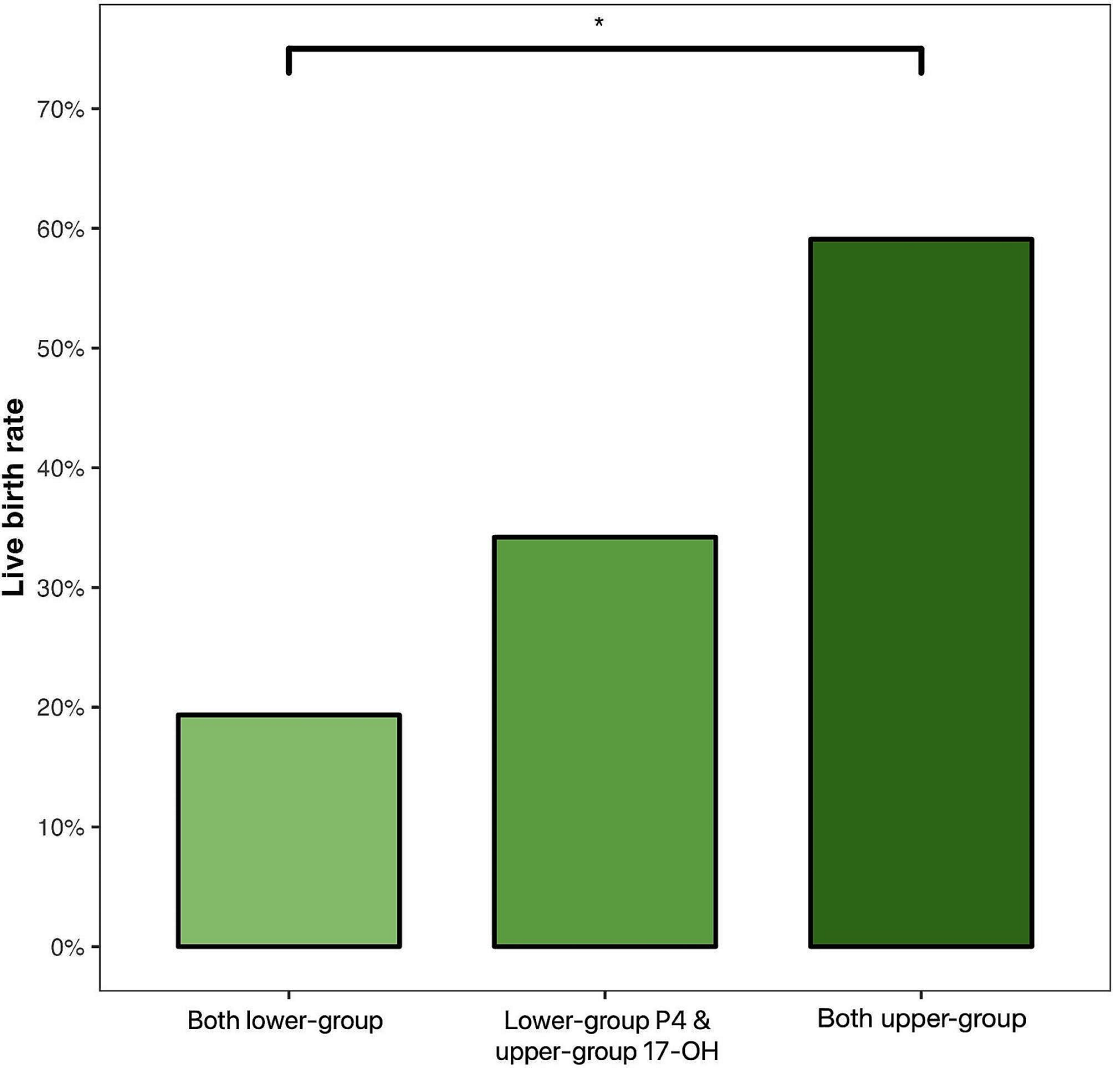
Combined influence of serum progesterone (P4) and 17-hydroxyprogesterone (17-OH-P4) profiles on the live birth rate (note: there were no cases with an upper serum progesterone profile and a lower 17-hydroxyprogesterone profile). *adjusted p-value < 0.05

Individuals with a lower profile for both serum progesterone and serum 17-hydroxyprogesterone had the lowest live birth rate (6/31, 19.4%) and those with an upper profile of both hormones had the highest live birth rate (13/22, 59.1%); there was an intermediate live birth rate in women with a low profile of serum progesterone and an upper profile of 17-hydroxyprogesterone, and no participants had a lower 17-hydroxyprogesterone/upper progesterone profile (Fig. 3).

Live birth occurred in 19/69 women with a lower progesterone profile (27.5%) and 13/22 with an upper progesterone profile (59.1%) (risk ratio 2.14; 95% CI 1.28 to 3.60). For serum 17-hydroxyprogesterone, live birth occurred in 6/31 women with a lower profile (19.4%) and 26/60 with an upper profile (43.3%) (risk ratio 2.24; 95% CI 1.03 to 4.86) (Fig. 2).

Live birth rates were highest when peak serum progesterone level occurred four or five days after oocyte pick-up (Table S5), and when serum 17-hydroxyprogesterone peaked at three or four days after oocyte pick-up (Table S6).

### Factors associated with with lower early luteal phase hormonal profiles

On multivariate analysis, factors associated with a lower progesterone profile were the anti-Müllerian hormone level, number of oocytes, number of day-3 embryos and number of good day-3 embryos (Table 2). Factors associated with a lower 17-hydroxyprogesterone profile on multivariate analysis were number of follicles of ≥ 14 mm and number of good day-3 embryos (Table 3). For both serum progesterone and 17-hydroxyprogesterone, the number of good day-3 embryos transferred was significantly associated with a lower hormone profile on univariate analysis, but not multivariate analysis (Tables 2 and 3).

## Discussion

To our knowledge, this study is the first to associate detailed information about the early luteal phase levels of progesterone and 17-hydroxyprogesterone with live birth in infertile women with normal ovarian reserve undergoing IVF with fresh embryo transfer. The live birth rate in those who had an upper profile of serum progesterone and serum 17-hydroxyprogesterone based on the AUC of measurements from the time of hCG trigger until implantation was three times higher than that in women with a lower serum profile of both hormones.

We believe that the analysis of the association between values that make up the overall luteal phase hormone profiles and the live birth rate is more meaningful than associations between the hormone level at each time point and the live birth rate because one woman may have hormone levels that reflect a lower profile at one timepoint and an upper profile at another timepoint whereas the overall total luteal phase hormonal profile for each woman provides better information about hormonal level exposure and therefore better information to evaluate for correlation with the live birth rate. Next, we identified factors that were associated with a lower hormone profile across the total luteal phase found that AMH, number of oocytes, day-3 embryos and good day-3 embryos were predictors for a lower profile of serum progesterone (Table 2), and the number of follicles of ≥ 14 mm and the number of good day-3 embryos were associated with a lower profile of 17-hydroxyprogesterone (Table 3).

Unlike protocols for ovarian stimulation during the follicular phase (which have undergone several refinements over the last 20–30 years), there is not currently any individualization of exogenous luteal phase support after use of a bolus hCG trigger during IVF. One contributing factor may be a relative lack of information about the relationship between early luteal phase hormone levels and fertility outcomes. Our group previously evaluated the early luteal phase hormonal profile after hCG trigger [4], and found a comparable serum progesterone trajectory to that in the current study, with considerable variability in the timing of peak progesterone between individuals. Our previous data also showed marked inter-individual variation in hCG concentrations after hCG trigger [4]. However, that study was conducted in the setting of frozen embryo transfer so the influence of early phase luteal hormone levels on fertility outcomes could not be determined.

The underlying mechanism for the marked difference in pregnancy rates between the lower and upper profile groups was not determined in the current study. Both groups were similar with respect to several participant characteristics but differed significantly in the number of follicles and oocytes collected, with the upper group having significantly higher numbers. Previous studies have shown that an increase in the number of oocytes aspirated during IVF treatment is associated with improved chances of pregnancy until a plateau is reached [7]. Having more oocytes/embryos available to select from in terms of embryo transfer clearly is beneficial, but it is not clear whether, for instance, a blastocyst with equal developmental competence and morphology score (potentially also tested for normal chromosomal status) has a better chance of implantation when it comes from a larger versus smaller cohort of embryos. The current study suggests that endometrial receptivity in the subgroup with an upper serum progesterone profile may be positively affected by the higher progesterone concentration provided by a higher number of corpora lutea compared with the lower serum progesterone profile group. It is likely that the endometrium cannot distinguish between progesterone produced from a few follicles versus many follicles, but the between-group difference in numbers of follicles and oocytes collected could reflect other factors that may influence the outcome of IVF such as advanced age.

Almost one in five women in this study had a very poor luteal phase despite the administration of 6500 IU hCG, which was characterized by early peak progesterone and insufficient progesterone secretion. The hCG bolus trigger provides a strong luteotropic stimulus and concentrations of progesterone rise a lot faster after an hCG bolus trigger than in natural cycles, but the progesterone peak after hCG trigger occurs earlier than in natural cycles. Individuals with a poor luteal phase after hCG trigger are not currently recognized in the clinic and, despite having good-quality embryos, the luteal phase is not sufficient to support pregnancy. It is therefore important to identify and detect these women.

Our data showed that the percentage increase from hCG trigger to oocyte pick-up was greater for 17-hydroxyprogesterone than for progesterone, that early levels were a better predictor of later luteal phase levels for 17-hydroxyprogesterone than for progesterone, and that endogenous production of 17-hydroxyprogesterone peaked about 12 h earlier than progesterone. This means that the endometrium is slightly more advanced than previously thought based on progesterone measurements. Early markers of the subsequent luteal phase need to be determined to allow hormone level profiles to be changed to a trajectory associated with a higher chance of conception in the lower hormone profile group. We found that concentrations of 17-hydroxyprogesterone measured at 24 h after induction of final follicular maturation were a predictor of later luteal phase hormone levels in the lower hormone profile group.

From a clinical perspective, it would be useful to define an early progesterone or 17-hydroxyprogesterone level below which additional luteal phase support could be considered after oocyte pick-up. However, our data do not allow this to be determined, and further research is needed to investigate approaches to altering the post-hCG trigger trajectory of progesterone/17-hydroxyprogesterone levels in a way that enhances the chance of conception. Nevertheless, determination of serum levels of these hormones early after hCG trigger would be required to allow individualization of luteal phase support, and this represents a change from the vast majority of current clinical practice.

Our results were similar for both serum progesterone and serum 17-hydroxyprogesterone, suggesting that endogenous hormone production by the corpus luteum is the important parameter in improving reproductive outcomes. Therefore, to increase pregnancy rates, the aim would be to secure better hormone secretion from the corpus luteum rather than relying on exogenous progesterone supplementation. In addition, the importance of both progesterone and 17-hydroxyprogesterone as predictors of conception in our study could reflect the fact that optimal function of both the luteinized granulosa and theca cells (producing progesterone and 17-hydroxyprogesterone, respectively) would help to improve the chances of achieving pregnancy during IVF. However, it is interesting that women in the lower serum hCG profile group had a slightly, but not significantly higher live birth rate compared with those in the upper hormone profile group. As the lower and upper group of patients show similar BMI and have received a similar bolus of hCG, the difference is likely to reflect either an enhanced clearance or an augmented consumption. However, given that those who conceive show higher levels of progesterone, it is hypothesized that hCG is faster consumed in the good prognosis group due to the higher number of corpora lutea, which collectively will contain more cells and express more LH receptors (LHR) that will bind and internalize hCG. In addition, this may reflect that each cell in the corpora lutea expresses an increased number of LHR, which in culture experiments have shown to result in enhanced progesterone secretion [8]. Furthermore, it is interesting that the 17-OH-P4 is higher in the upper group already from the hCG trigger compared to the lower group, which is not the case for progesterone.

Collectively, these observations further indicate that hormonal interactions between hCG and the corpus luteum are important for achieving pregnancy, and therefore additional studies are needed to determine the reasons for reduced progesterone and 17-hydroxyprogesterone production in those with a lower serum profile of hCG. Women in our study who had a peak progesterone level at four days or later after oocyte pick-up had a live birth rate that was more than twice that in women with an earlier serum progesterone peak. In fact, more than 82% of all live births in the current study occurred in women whose serum progesterone/17-hydroxyprogesterone level peaked on the fourth or fifth day after oocyte pick-up. In addition, those with an early progesterone/17-hydroxyprogesterone peak had higher rates of early pregnancy loss. Half of the pregnancy loss before 12 weeks of gestation occurred in individuals who had peak progesterone level on or before the second day after oocyte pick-up. Based on this study and our previous work [4], women with a peak progesterone on or before the third day after oocyte pick-up comprise about 15–20% of the IVF population. This is not a small proportion, and it is important to identify these individuals early so that luteal phase support can be increased. Given that the best chance for live birth occurred when progesterone peaked on day four after oocyte pick-up, prompt intervention would be required to allow exogenous hormone supplementation that provides peak levels in the optimal time period.

Given the significantly higher live birth rate in individuals in the upper progesterone profile group, an important question is how to ensure that the majority of women fall into this group and therefore have a better chance of achieving pregnancy and live birth. One approach could be to use additional low-dose hCG during the early to mid-luteal phase, potentially rescuing endometrial receptivity. However, some women may not respond with increased progesterone and 17-hydroxyprogesterone output and may require exogenous progesterone. This needs to be evaluated in appropriately designed clinical studies.

A key strength of this study is that it addresses an important knowledge gap regarding associations between early luteal phase hormone levels and fertility outcomes in women undergoing IVF with fresh embryo transfer. The study had a prospective design with respect to collection of early phase luteal hormone levels, but associations between these and the live birth rate represent correlations without providing direct evidence of causality. With respect to serum hormone levels, we cannot exclude the possibility that serum progesterone levels might vary due to inter-individual differences in the absorption of vaginal progesterone provided as luteal phase support, although the influence should be similar in both post hoc defined groups. In addition, our data do not allow us to categorically determine whether the timing of the progesterone peak or the absolute progesterone concentration make the greatest contribution to the chances of achieving pregnancy/live birth; this is an area for future study. Finally, the external generalizability of the study findings is limited by the nature of the study population (all were from Vietnam).

## Conclusion

This study provides detailed information about early luteal phase hormone profiles and evaluated their association with the live birth rate for the first time. There is huge potential to develop luteal phase support approaches that ensure sufficient output of key hormones in the optimal time window to increase pregnancy and live birth rates in individuals undergoing IVF with fresh embryo transfer.

### Electronic supplementary material

Below is the link to the electronic supplementary material.

Supplementary Material 1

## Data Availability

Access to relevant anonymized patient level data will be considered on reasonable request submitted to the corresponding author.

## Declarations

### Transparency declaration

The lead author affirms that this manuscript is an honest, accurate, and transparent account of the study being reported. The reporting of this work is compliant with STROBE guidelines. The lead author affirms that no important aspects of the study have been omitted and that any discrepancies from the study as planned. The IVFMD, My Duc Hospital, Vietnam Ethic Review Board approved this study (approval number: 18/20/DD-BVMD). This prospective cohort study was registered at www.clinicaltrials.gov on 28 December 2020 (registration number: NCT04693624).

## Abbreviations

CI: Confidence interval
hCG: Human chorionic gonadotropin
IVF: In vitro fertilization
RR: Risk ratio
