# Supplementary material for: In vitro fertilization outcome based on the detailed early luteal phase trajectory of hormones: a prospective cohort study
Source: Reprod Biol Endocrinol. 2024 May 20;22:56. doi: 10.1186/s12958-024-01229-3 (PMC11103990; doi:10.1186/s12958-024-01229-3)
Supplement: Supplementary file 1 — Supplementary Material 1 [file 12958_2024_1229_MOESM1_ESM.doc]

**SUPPLEMENTARY MATERIAL**

**In vitro fertilization outcome based on the detailed early luteal phase trajectory of hormones: a prospective cohort study**

Lan N Vuong MD PhD^1,2^ Toan D Pham MSc,^2,3^ Vu N A Ho MD,^2,3^ Anh T L Vu BSc,^2,3^ Tuong M Ho MD,^2,3^ Claus Yding Andersen MSc DMSc^4^

^1^Department of Obstetrics and Gynaecology, University of Medicine and Pharmacy at HCMC, Ho Chi Minh City, Vietnam

^2^IVFMD, My Duc Hospital, Ho Chi Minh City, Vietnam

^3^HOPE Research Center, My Duc Hospital, Ho Chi Minh City, Vietnam

^4^Institute of Clinical Medicine, The Faculty of Health Science, Copenhagen University, Copenhagen, Denmark

**Supplementary Tables**

**Table S1** Hormone levels over time

| **Time points**† | **Progesterone, nmol/L** | **17-hydroxyprogesterone, nmol/L** | **Beta-hCG, mIU/mL** |
| --- | --- | --- | --- |
| Before hCG | 0.96±0.21 | 8.8±0.7 | 2.1±0.6 |
| hCG + 12 hours | 16.2±1.1 | 22.6±1.0 | 121.6±5.9 |
| hCG + 24 hours | 34.2±5.1 | 29.4±1.2 | 126.2±5.5 |
| hCG + 36 hours | 35.2±3.5 | 27.7±1.1 | 114.4±4.2 |
| Oocyte pick-up + 1 day | 143.1±6.2 | 32.7±1.3 | 64.7±2.3 |
| Oocyte pick-up + 2 days | 227.7±9.1 | 39.1±1.4 | 37.2±1.4 |
| Oocyte pick-up + 3 days | 307.5±11.8 | 43.9±1.3 | 18.8±0.8 |
| Oocyte pick-up + 4 days | 365.7±15.8 | 44.1±1.6 | 9.9±0.5 |
| Oocyte pick-up + 5 days | 332.7±16.0 | 40.5±1.6 | 5.2±0.3 |
| Oocyte pick-up + 6 days | 256.7±14.9 | 29.8±1.6 | 3.1±0.2 |

Values are mean ± standard deviation.

hCG, human chorionic gonadotropin.

†Data for Before hCG were available in 94 participants, and Oocyte pick-up + 6 days in 92 participants; all other time points had data available for all 95 participants.

**Table S2** Hormone levels over time in individuals with in the lower versus upper profile groups

| **Time points** | **Progesterone,  nmol/L** | | **17-hydroxyprogesterone, nmol/L** | |
| --- | --- | --- | --- | --- |
|  | **Lower profile group (n=69)** | **Upper profile group (n=22)** | **Lower profile group (n=31)** | **Upper profile group (n=60)** |
| Before hCG | 3.1±5.9 | 2.0±1.2 | 6.4±0.9 | 10.2±1.0 |
| hCG + 12 hours | 14.6±1.2 | 22.2±2.0 | 15.6±1.0 | 26.5±1.2 |
| hCG + 24 hours | 24.5±1.4 | 45.5±3.6 | 19.2±1.3 | 35.0±1.4 |
| hCG + 36 hours | 28.8±3.0 | 51.9±10.2 | 19.0±1.2 | 32.4±1.2 |
| Oocyte pick-up + 1 day | 120.6±4.7 | 207.0±14.5 | 22.4±2.0 | 38.1±1.2 |
| Oocyte pick-up + 2 days | 192.3±7.7 | 330.3±17.3 | 26.4±1.9 | 45.6±1.3 |
| Oocyte pick-up + 3 days | 259.2±9.8 | 453.4±16.8 | 30.2±1.8 | 51.0±0.8 |
| Oocyte pick-up + 4 days | 300.8±12.8 | 572.6±22.3 | 26.3±2.3 | 53.3±1.0 |
| Oocyte pick-up + 5 days | 274.7±13.5 | 526.6±27.2 | 25.8±1.9 | 48.5±1.4 |
| Oocyte pick-up + 6 days | 208.0±12.2 | 406.3±32.6 | 19.2±2.0 | 35.5±1.9 |

Values are mean ± standard deviation.

hCG, human chorionic gonadotropin.

**Table S3.** Correlation between serum progesterone, 17-hydroxy progesterone and beta hCG levels at each time point and the live birth rate (adjusted for the anti-Müllerian hormone level)

|  | **Adjusted OR [95% CI], p-value for the association between each hormone level and live birth** | | |
| --- | --- | --- | --- |
|  | **Progesterone** | **17-hydroxyprogesterone** | **Beta hCG** |
| Before hCG | 0.99 [0.94, 1.00]; 0.317 | 1.00 [0.93, 1.06]; 0.94 | 0.80 [0.55, 1.12]; 0.210 |
| hCG + 12 hours | 1.00 [0.96, 1.05]; 0.871 | 1.04 [0.99, 1.10]; 0.132 | 1.00 [0.99, 1.01]; 0.598 |
| hCG + 24 hours | 1.00 [0.97, 1.03]; 0.937 | 1.01 [0.97, 1.06]; 0.516 | 1.00 [0.99, 1.01]; 0.627 |
| hCG + 36 hours | 0.99 [0.97, 1.01]; 0.293 | 0.99 [0.97, 1.00]; 0.274 | 1.00 [0.98, 1.01]; 0.582 |
| Oocyte pick-up + 1 day | 1.00 [0.99, 1.01]; 0.773 | 1.00 [0.96, 1.04]; 0.925 | 1.00 [0.97, 1.02]; 0.692 |
| Oocyte pick-up + 2 days | 1.00 [1.00, 1.01]; 0.458 | 1.01 [0.97, 1.04]; 0.701 | 0.99 [0.95, 1.02]; 0.510 |
| Oocyte pick-up + 3 days | 1.00 [1.00, 1.01]; 0.353 | 1.02 [0.98, 1.06]; 0.404 | 1.00 [0.94, 1.06]; 0.950 |
| Oocyte pick-up + 4 days | 1.00 [1.00, 1.01]; 0.242 | 1.00 [0.97, 1.03]; 0.964 | 1.00 [0.91, 1.09]; 0.994 |
| Oocyte pick-up + 5 days | 1.00 [1.00, 1.01]; 0.207 | 1.02 [0.99, 1.06]; 0.243 | 1.01 [0.87, 1.17]; 0.874 |
| Oocyte pick-up + 6 days | 1.00 [1.00, 1.00]; 0.824 | 1.00 [0.97, 1.03]; 0.891 | 0.96 [0.77, 1.18]; 0.728 |
| Profile (lower vs. upper) | 0.29 [0.10, 0.85]; 0.025 | 0.36 [0.11, 1.02]; 0.063 | 1.61 [0.67, 3.95]; 0.291 |

CI, confidence interval; hCG, human chorionic gonadotropin; OR, odds ratio.

**Table S4.** Correlation between levels of serum progesterone, 17-hydroxyprogesterone and beta-human chorionic gonadotropin levels at each time point and the live birth rate (adjusted for the number of good embryos transferred)

|  | **Adjusted OR [95% CI]** | | |
| --- | --- | --- | --- |
|  | **Progesterone** | **17-hydroxyprogesterone** | **Beta hCG** |
| Before hCG | 0.99 [0.94, 1.00]; 0.302 | 0.99 [0.92, 1.05]; 0.742 | 0.82 [0.57, 1.15]; 0.272 |
| hCG + 12 hours | 1.00 [0.95, 1.04]; 0.932 | 1.04 [0.99, 1.09]; 0.119 | 1.00 [0.99, 1.01]; 0.946 |
| hCG + 24 hours | 1.00 [0.97, 1.03]; 0.916 | 1.02 [0.98, 1.06]; 0.403 | 1.00 [0.99, 1.01]; 0.865 |
| hCG + 36 hours | 0.99 [0.96, 1.01]; 0.291 | 0.99 [0.95, 1.04]; 0.731 | 1.00 [0.99, 1.01]; 0.971 |
| Oocyte pick-up + 1 day | 1.00 [0.99, 1.01]; 0.955 | 1.00 [0.96, 1.04]; 0.894 | 1.00 [0.98, 1.02]; 0.793 |
| Oocyte pick-up + 2 days | 1.00 [1.00, 1.01]; 0.422 | 1.01 [0.97, 1.05]; 0.661 | 1.00 [0.97, 1.03]; 0.958 |
| Oocyte pick-up + 3 days | 1.00 [1.00, 1.01]; 0.267 | 1.02 [0.98, 1.07]; 0.316 | 1.02 [0.96, 1.08]; 0.494 |
| Oocyte pick-up + 4 days | 1.00 [1.00, 1.01]; 0.142 | 1.00 [0.97, 1.03]; 0.828 | 1.02 [0.93, 1.11]; 0.719 |
| Oocyte pick-up + 5 days | 1.00 [1.00, 1.01]; 0.102 | 1.02 [0.99, 1.06]; 0.127 | 1.03 [0.89, 1.19]; 0.702 |
| Oocyte pick-up + 6 days | 1.00 [1.00, 1.00]; 0.406 | 1.01 [0.99, 1.04]; 0.338 | 1.01 [0.80, 1.25]; 0.929 |
| Profile (lower vs upper) | 0.34 [0.12, 0.93]; 0.038 | 0.41 [0.13, 1.16]; 0.103 | 1.46 [0.60, 3.65]; 0.408 |

CI, confidence interval; hCG, human chorionic gonadotropin; OR, odds ratio.

**Table S5.** Subgroup analysis: timing of peak progesterone level and rates of live birth and miscarriage*

|  | **Time of peak,  no. (%)**† | **Serum progesterone** | | **Live birth, no. (%)** | **Miscarriage at <12 weeks, n (%)** | **Miscarriage at 12 to <24 weeks, n (%)** |
| --- | --- | --- | --- | --- | --- | --- |
|  |  | **Peak, nmol/L** | **[Peak value–value at OPU+6]/peak, %** |  |  |  |
| hCG + 12 hours | 0 (0) | - | - | - | - | - |
| hCG + 24 hours | 0 (0) | - | - | - | - | - |
| hCG + 36 hours | 0 (0) | - | - | - | - | - |
| Oocyte pick-up + 1 day | 0 (0) | - | - | - | - | - |
| Oocyte pick-up + 2 days | 1 (1.1) | 334.48 | 79.08 | 0/1 (0) | 0/1 (0) | 0/1 (0) |
| Oocyte pick-up + 3 days | 11 (12.09) | 304.86±173.61 | 58.76±16.9 | 3/11 (27.3) | 0/11 (0) | 0/11 (0) |
| Oocyte pick-up + 4 days | 49 (53.85) | 410.62±150.16 | 42.34±20.51 | 19/49 (38.78) | 5/49 (10.2) | 0/49 (0) |
| Oocyte pick-up + 5 days | 19 (20.88) | 436.44±140.3 | 23.59±14.4 | 7/19 (36.84) | 0/19 (0) | 0/19 (0) |
| Oocyte pick-up + 6 days | 10 (10.99) | 369.77±151.81 | 0 | 3/10 (30) | 2/10 (20) | 0/10 (0) |

*Plus-minus values are mean ± standard deviation. †Of a total 91 participants.

**Table S6.** Subgroup analysis: timing of peak 17-hydroxyprogesterone level and rates of live birth and miscarriage

|  | **Time of peak, n (%)**† | **Serum 17-hydroxyprogesterone** | | **Live birth, n (%)** | **Miscarriage at <12 weeks, n (%)** | **Miscarriage at 12 to <24 weeks, n (%)** |
| --- | --- | --- | --- | --- | --- | --- |
|  |  | **Peak, nmol/L** | **[Peak value–value at OPU+6]/peak, %** |  |  |  |
| Before hCG | 0 (0) | - | - | - | - | - |
| hCG + 12 hours | 0 (0) | - | - | - | - | - |
| hCG + 24 hours | 1 (1.1) | 39.67 | 2.59 | 0/1 (0) | 1/1 (100.0) | 0/1 (0) |
| hCG + 36 hours | 0 (0) | - | - | - | - | - |
| Oocyte pick-up + 1 day | 3 (3.3) | 49.66±23.89 | 42.57±37.45 | 1/3 (33.3) | 0/3 (0) | 0/3 (0) |
| Oocyte pick-up + 2 days | 9 (10.0) | 50.95±11.61 | 56.69±28.08 | 2/9 (22.2) | 3/9 (33.3) | 0/9 (0) |
| Oocyte pick-up + 3 days | 20 (22.2) | 47.23±12.72 | 59.69±26.18 | 10/20 (50.0) | 0/20 (0) | 0/20 (0) |
| Oocyte pick-up + 4 days | 33 (36.7) | 52.71±12.34 | 38.49±18.23 | 10/33 (30.3) | 2/33 (6.1) | 0/33 (0) |
| Oocyte pick-up + 5 days | 19 (21.1) | 48.5±11.5 | 27.77±20.25 | 7/19 (36.8) | 1/19 (5.3) | 0/19 (0) |
| Oocyte pick-up + 6 days | 6 (6.7) | 43.38±15.49 | 0 | 2/6 (33.3) | 0/6 (0) | 0/6 (0) |

Values are mean ± standard deviation or number of participants (%).

†Of a total 91 participants.
